# EXAMINING AND ADDRESSING BARRIERS TO MENTAL HEALTH CARE FOR OLDER ADULTS IN PERMANENT SUPPORTIVE HOUSING

**DOI:** 10.1093/geroni/igae098.0397

**Published:** 2024-12-31

**Authors:** Randee Robinson

**Affiliations:** University of Washington, Seattle, Washington, United States

## Abstract

Permanent supportive housing is an evidence-based intervention to support individuals exiting from chronic homelessness. Increasingly, the demographic of adults prioritized for PSH are over the age of 50. Despite the higher prevalence of mental health concerns amongst older PSH residents their engagement with mental health services remains limited. The purpose of this investigation was to examine barriers to mental health care for older adults living in PSH and provide suggestions to address the identified barriers. A one-hour semi-structured focus group protocol was developed. Question sets focused on individual and system level barriers to mental health services. The focus group was digitally recorded, and responses were transcribed. Eight PSH residents from one housing provider participated in the focus group session. Thematic analysis utilizing inductive and deductive approaches was completed by two individuals. Themes of case manager role and limitations, stigma, service navigation, and trustworthiness emerged from the data. The mean age of participants was 63.8 years (SD 9.78). The mean length of tenancy was 6.6 years (SD 3.29). Addressing mental health access is critically important for older adults in PSH. Interventions to improve mental health care access at both the individual and systems level should target building trust, reinforcing available services, clarifying the care manager’s role, and supporting staff consistency. Future research should target effective interventions to address these barriers.

